# The Deformation Rate of Smooth Muscle Cells in Vessel Walls After Short-Duration Heating Dilatation in a Porcine Model *Ex Vivo* and *In Vivo*

**DOI:** 10.1007/s13239-012-0093-7

**Published:** 2012-05-08

**Authors:** Mie Kunio, Tsunenori Arai

**Affiliations:** 1 School of Fundamental Science and Technology, Graduate School of Science and Technology, Keio University, 3-14-1, Hiyoshi, Kohoku-ku, Yokohama, Kanagawa 223-8522 Japan; 2 Department of Applied Physics and Physico-Informatics, Faculty of Science and Technology, Keio University, Yokohama, Kanagawa Japan

**Keywords:** Balloon angioplasty, Thermal angioplasty, Smooth muscle cell, Deformation, Arterial dilatation

## Abstract

We have proposed a novel short-duration thermal angioplasty with uniform temperature distribution. Although the dilatation mechanism of our short-duration heating dilatation was reported in our previous study, the influences on smooth muscle cells (SMCs) are not sufficiently understood. We studied the influences on SMCs in terms of shape change and discussed the relationship between the SMCs’ shape change and dilatation mechanism *ex vivo* and *in vivo*. We found that the SMCs were fixed in the stretched condition after our short-duration heating dilatation both *ex vivo* and *in vivo*. The deformation rate of SMCs’ shape, measured by the cells’ nuclei, was increased with rising balloon maximum temperature (*T*_balloon_), and the same tendency was observed for the arterial dilatation rate. We hypothesize that the SMCs were fixed in the stretched condition because the arterial dilatation with our short-duration heating dilatation was performed without any plastic deformations of the vessel wall, causing the vessel wall itself to be stretched. We also prospect that the reasons for the positive correlation between the deformation rate of SMCs’ shape and *T*_balloon_ are that (i) the area heated over 60 °C was expanded with rising *T*_balloon_, and (ii) the arterial dilatation rate was also increased with rising *T*_balloon_.

## Introduction

Percutaneous transluminal angioplasty (PTA) is the conventional treatment to get revascularization of arterial stenosis.^16^ Since plastic deformation of the vessel wall is necessary to obtain sufficient arterial dilatation with PTA immediately after the procedure, vascular injuries in the vessel wall are unavoidable. Smooth muscle cells (SMCs) migrate and proliferate excessively as a repair response of these vascular injuries,^18^ resulting in the neo-intimal hyperplasia formation on the chronic phase. Stent implantation was developed to sustain arterial dilatation with PTA until the chronic phase. However, this procedure is not used commonly in peripheral artery region^16^ due to the frequent occurrence of stent fracture. Therefore, a new method is needed to improve the performances of PTA.

In the late 1980s, thermal angioplasty^3^,^23^ was developed as a new method to dilate arteries without any vascular injuries which were induced by the plastic deformation with PTA. With the thermal angioplasty, the mechanical characteristics of the vessel wall were changed by the temperature rise of the vessel wall during heating dilatation. This method used collagen thermal denaturation to soften the vessel wall. Although sufficient arterial dilatations were obtained on the acute phase, complete occlusions were observed in most cases on the chronic phase due to serious thermal damages to the adventitia and surrounding tissues.

We have proposed a novel short-duration thermal angioplasty with uniform temperature distribution, Photo-thermo Dynamic Balloon Angioplasty^20^,^21^ to reduce thermal damages to the adventitia and surrounding tissue, which were the major cause of restenosis after other thermal angioplasties. Short-duration heating (<15 s, <70 °C) is realized by the combination of laser light irradiation and continuous constant fluid flow inside the balloon. The temperature at the vessel wall and heating time are arranged by the laser light power and irradiation time. Low dilatation pressure (<0.4 MPa) can be used in our short-duration heating dilatation because the vessel wall becomes softened by heating. We have obtained sufficient arterial dilatation with short-duration heating and low dilatation pressure on both acute and chronic phase in our previous *in vivo* canine and rabbit studies.^2^,^14^,^24^ The mechanism of our short-duration heating dilatation have been reported^21^; (i) reversible collagen thermal denaturation and stretch-fixing of elastin occur during short-duration heating balloon dilatation, and (ii) collagen fiber orients circumferentially and reconstructs with the temperature decrease after short-duration heating. Therefore, the arterial dilatation with our short-duration heating dilatation was performed without any vascular injuries, which are the main causes of the restenosis after the balloon angioplasty. Although the dilatation mechanism has been well understood, the influences on SMCs, which play the most important role on chronic performances, are not investigated yet. To understand the relationship between the influences on SMCs and the dilatation mechanism, we studied the influences on SMCs with our short-duration heating dilatation in terms of the SMCs’ shape change by *ex vivo* experiments using healthy porcine arteries and *in vivo* chronic porcine experiments.

## Materials and Methods

### Prototype Balloon Catheter and Equipment for Short-Duration Heating Dilatation

**Figure 1:**
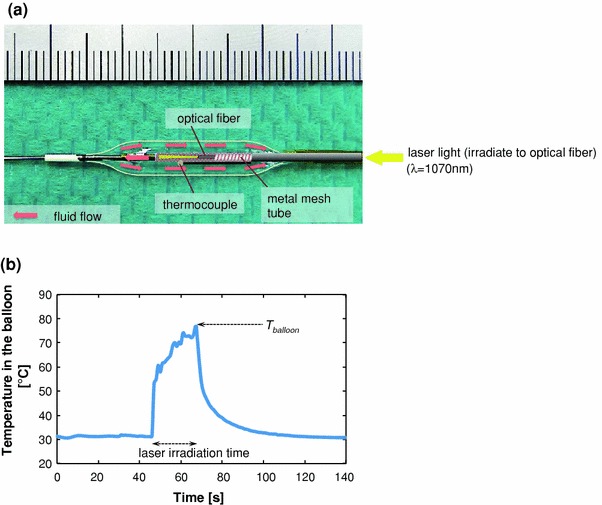
(a) The schematic structure of the prototype short-duration heating balloon catheter; (b) the typical temperature history inside the balloon

Figure 1a shows the schematic structure of the prototype short-duration heating balloon catheter. The laser light (neodymium-doped fiber laser FL-30-2; λ = 1070 nm, Cyber Laser, Tokyo, Japan) was delivered into the metal mesh tube through an optical fiber. The laser light energy was transformed to heat energy by multiple reflections inside the metal mesh tube. The generated heat was transferred to the irrigated contrast media which flowed in the gap between the metal mesh tube and optical fiber. The heated contrast media was flowed in the balloon continuously at a rate of 0.03–0.06 mL/s to get uniform temperature distribution throughout the balloon. The balloon pressure was maintained at 0.35 MPa by specially designed irrigation equipment. The balloon temperature was controlled by the laser light power measuring its temperature by a thermocouple positioned at the center of balloon. The laser light was irradiated for around 15 s. A typical temperature history inside the balloon is shown in Fig. 1b. The rapid increase and decrease of temperature in the balloon was realized by the irrigation equipment mentioned above. The balloon temperature reached the maximum just before the laser light being turned off. We defined this maximum temperature in the balloon during short-duration heating dilatation as *T*_balloon_. We also defined the laser light irradiation time as heating time. The balloon temperature decreased and became less than 37 °C within 60 s from the laser light being turned off.

### *Ex Vivo* Experiment

Porcine carotid arteries were used within 5 h of extraction. To relax the vessel wall, 1.0 × 10^−6^ mol/L papaverine solution (10% papaverine hydrochloride powder; Maruishi, Osaka, Japan), diluted with Tyrode solution, was perfused gently into the arteries for 20 min. After this procedure, the arterial inner diameter was 1.46 ± 0.05 mm and the media thickness was 0.54 ± 0.12 mm. To simulate the dimension of blood flow direction inside the body, the arteries were stretched 1.2-fold in length. The stretched arteries were placed in a constant-temperature bath (37 °C) with Tyrode solution during dilatations. The prototype short-duration heating balloon catheter (non-compliant, diameter: 3.0 mm at 0.35 MPa in balloon pressure, effective length: 20 mm) and the conventional balloon catheter (semi-compliant, diameter: 3.14 mm at 1.0 MPa, 3.32 mm at 1.5 MPa in balloon pressure, effective length: 20 mm) were used to dilate the fresh carotid arteries. The performed short-duration heating dilatation conditions were as follows; 60–75 °C in *T*_balloon_, 15 s in heating time, 0.35 MPa in balloon pressure. The performed conventional balloon dilatation conditions were as follows; 1.0–1.5 MPa in balloon pressure, 60 s in dilatation time. Immediately after dilatations, the arteries were fixed with 10% formalin solution, with length maintained at 1.2-fold and inner pressure free. Hematoxylin–eosin (HE) stained specimens were made from these arteries.

### *In Vivo* Experiment

*In vivo* porcine study was performed according to the principles of the Declaration of Helsinki and was approved by the ethical committee of Keio University in Japan. Six female pigs (age: 3–3.5 months, weight: 30–35 kg, breed: LWD, Japan) were anesthetized by isoflurane and ketamine. Their heart rate, arterial pressure, and deep body temperature were continuously monitored. A 7 Fr. vascular sheath (Radiofocus Introducer II Standard kit; Terumo, Tokyo, Japan) was placed in the left carotid artery. A 7 Fr. guiding catheter (Autobahn soft MP; Nipro, Osaka, Japan) and 0.018″ guide wire (ABYSS; Nipro, Osaka, Japan) were inserted through the vascular sheath. After the administration of 2 mg nitrol to the arteries for relaxation of the vessel wall, an angiographic image was captured to measure the control diameter of both iliac and femoral arteries. The prototype short-duration heating balloon catheter (diameter: 5.5, 6.0, 6.5 mm at 0.35 MPa in balloon pressure, effective length: 20 mm) and the conventional balloon catheter (diameter: 5.5, 6.0, 6.5 mm at 1.0 MPa in balloon pressure, effective length: 20 mm) was selected, of which diameter was 1.3–1.5-fold of the measured arterial control diameter. The catheter was delivered to the target region using X-ray fluoroscopic image guiding. The performed short-duration heating dilatation conditions were as follows; 65, 75, and 85 °C (±5 °C, *N* = 6 respectively) in *T*_balloon_, 15–20 s in heating time, and approximately 0.35 MPa in balloon pressure. The performed conventional balloon dilatation conditions were as follows; 1.0 MPa in balloon pressure, and 60 s in dilatation time. Immediately after the arterial dilatations, the angiographic images were captured to check the arterial dilatation effect on acute phase. Four weeks after the dilatations, the pigs were killed by overdose of isoflurane and the dilated arteries were extracted. Soon after extractions, the arteries were fixed with 10% formalin solution simulating the dimension of blood flow direction inside the body. HE stained specimens were made from these arteries.

### Measurement Method for the Deformation Rate of SMCs’ Shape

According to report,^4^ the spread cells and isolated nuclei presented the same deformation when they were compressed between micro plates. The SMC is a kind of spread cell, and another report^15^ shows that the deformation of SMCs’ nuclei was same as that of SMCs during a quasi-in situ tensile test.

**Figure 2:**
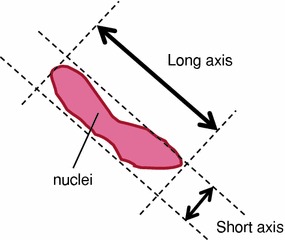
The measurement method for the long and short axes of SMCs’ nuclei

Microscopic images of the HE stained specimens were taken with a bright-field microscope (BX-51; Olympus, Tokyo, Japan) and digital camera (D5000; Nikon, Tokyo, Japan). Considering the shape of SMCs’ nuclei as an ellipse, the ratio of long and short axes was measured from the microscopic images (Fig. 2) with the image analysis software (ImageJ; National Institutes of Health, MD, USA). The long and short axes were defined by ourselves. We calculated the deformation rate of SMCs’ shape by dividing the ratio of long and short axes after dilatation by that before dilatation.

### Measurement Method for the Arterial Dilatation Rate

We measured the arterial dilatation rate to evaluate the dilatation effect.

In *ex vivo* experiments, the arterial inner diameters were measured by digital microscope (sz-7000; Scalar, Tokyo, Japan) with the HE stained specimens. The arterial dilatation rate was calculated by dividing the arterial inner diameter after dilatation by that before dilatation.

In *in vivo* experiments, the luminal areas were measured with ImageJ from the microscopic images of the HE stained specimens. The luminal area in which the dilatation was performed was divided by that in which the dilatation was not performed. The arterial dilatation rate was calculated from the square root of the result of this division.

## Results

### Histological Observations

**Figure 3:**
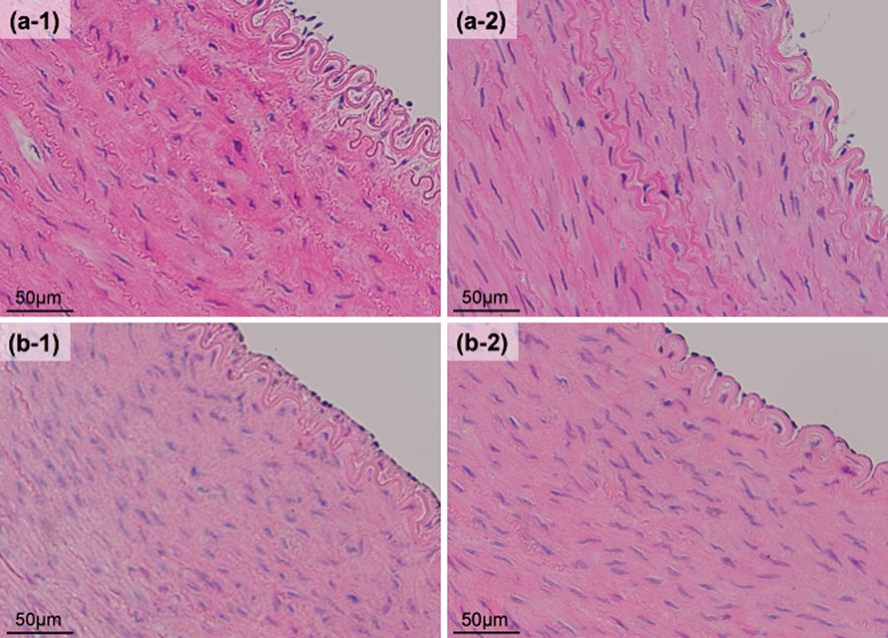
The bright-field microscopic images of the HE stained specimens: the arterial dilatations with our short-duration heating dilatation. (a-1) Before dilatation *ex vivo.* (a-2) After dilatation (75 °C in *T*_balloon_) *ex vivo.* (b-1) Not performing dilatation *in vivo* (1 month later). (b-2) Performing dilatation (75 °C in *T*_balloon_) *in vivo* (1 month later)

**Figure 4:**
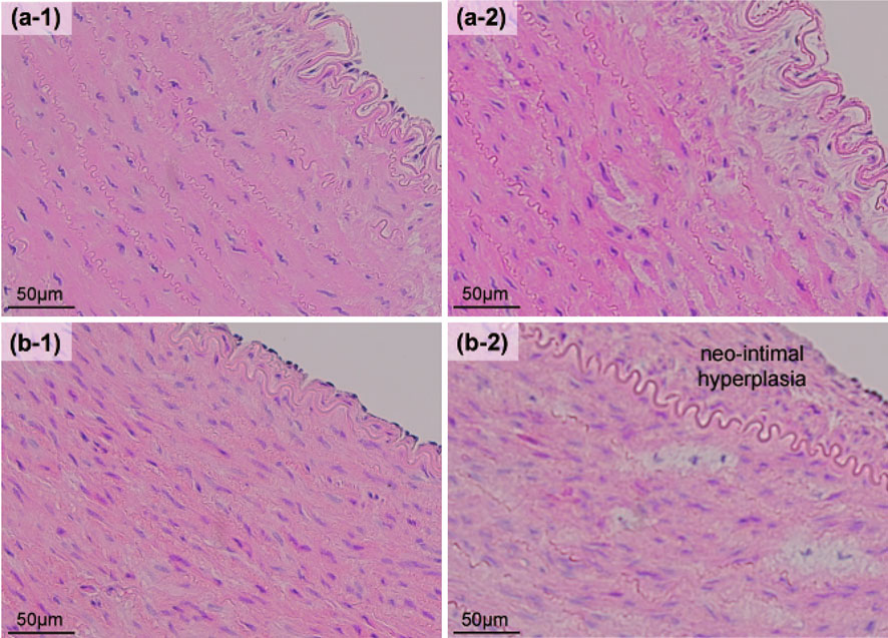
The bright-field microscopic images of the HE stained specimens: the arterial dilatations with the conventional balloon dilatation. (a-1) Before dilatation *ex vivo.* (a-2) After dilatation (1.0 MPa in balloon pressure) *ex vivo.* (b-1) Not performing dilatation *in vivo* (1 month later). (b-2) Performing dilatation (1.0 MPa in balloon pressure) *in vivo* (1 month later)

Bright-field microscopic images of the HE stained specimens are shown in Figs. 3 and 4. As shown in Fig. 3, the SMCs were fixed in the stretched condition after our short-duration heating dilatation both *ex vivo* and *in vivo*. The results indicate that the stretched condition of the SMCs was maintained until the chronic phase. The shape of the SMCs was not changed after the conventional balloon dilatation either *ex vivo* or *in vivo* (Fig. 4).

### Measurement Results: The Deformation Rate of SMCs’ Shape and the Arterial Dilatation Rate (*Ex Vivo* and *In Vivo*)

**Figure 5:**
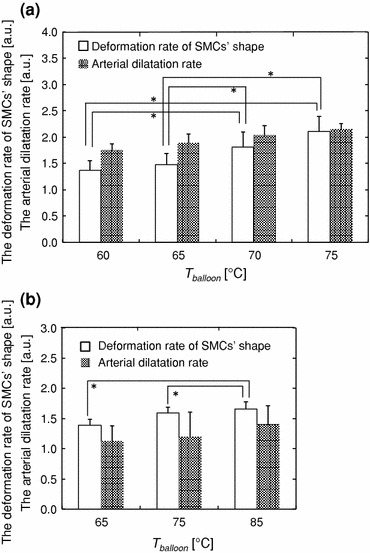
The relationship between *T*_balloon_, the deformation rate of SMCs’ shape and arterial dilatation rate. (a) Results of *ex vivo* experiment. (b) Results of *in vivo* experiment.* Significant differences (*p* < 0.05) exist from Student’s *t* test

The deformation rate of SMCs’ shape was measured to reveal the relationship between *T*_balloon_ and SMCs’ shape change. After our short-duration heating dilatation, the deformation rate of SMCs’ shape increased with rising *T*_balloon_ within 60–75 °C *ex vivo* and 65–85 °C *in vivo* (Fig. 5). The arterial dilatation rate was also increased with rising *T*_balloon_ both *ex vivo* and *in vivo* (Fig. 5). These results indicate that both the deformation rate and arterial dilatation rate are positively correlated with *T*_balloon_.

After the conventional balloon dilatation, the deformation rate of SMCs’ shape was almost 1.0 at any dilatation conditions *ex vivo*; 1.01 ± 0.14 at 1.0 MPa, 1.09 ± 0.18 at 1.15 MPa, 1.09 ± 0.18 at 1.35 MPa, and 1.01 ± 0.15 at 1.5 MPa in balloon dilatation pressure. No significant dependence of the deformation rate on balloon dilatation pressure was observed. On the other hand, the arterial dilation rate was increased with the balloon dilatation pressure *ex vivo*; 1.21 at 1.0 MPa, 1.25 at 1.15 MPa, 1.58 at 1.35 MPa, and 1.70 at 1.5 MPa. This change of the arterial dilatation rate is because the conventional balloon catheter was semi-compliant. The deformation rate of SMCs’ shape *in vivo* was 1.11 ± 0.12 and the arterial dilatation rate was 0.93 ± 0.17 at 1.0 MPa in balloon dilatation pressure.

## Discussions

### Relationship Between the Shape Change of the SMCs and Dilatation Mechanism

We found that the shape of SMCs was fixed in the stretched condition after our short-duration heating dilatation both *ex vivo* and *in vivo*. The deformation rate of SMCs’ shape increased with rising *T*_balloon_ both *ex vivo* and *in vivo*, and the same tendency was observed for the arterial dilatation rate. The arterial dilatation with the thermal angioplasty, including our short-duration heating dilatation, is performed while the vessel wall is softened by the collagen thermal denaturation, which starts at around 60 °C.^9^,^25^ The temperature in the vessel wall during our short-duration heating dilatation was estimated by thermal conduction calculation (detailed explanations are provided in our previous report^7^). The parameters which were used for the calculation are summarized in Table 1. The area heated over 60 °C was increased from 0.05 to 0.42 mm with rising *T*_balloon_ within 65–85 °C from our estimation results, and thus the softened area of the vessel wall expanded with rising *T*_balloon_. This meant that the Young’s modulus of the vessel wall decreased and that the maximum deformation volume of the vessel wall increased.^13^ We can obtain larger arterial dilatation without any plastic deformation of the vessel wall at the same dilatation pressure according with rising *T*_balloon_. Therefore, we hypothesize that the SMCs in the vessel wall were fixed in the stretched condition after our short-duration heating dilatation and that the deformation rate increased with rising *T*_balloon_.

**Table 1:** The parameters used for the thermal conduction calculation

	Density (g/mm^3^)	Conductivity (W/mm °C)	Specific heat* (J/g °C)	Initial temperature (°C)
Arterial wall	1.0 × 10^−3^	4.2 × 10^−4^	5.0 (< 40 °C)	37
			5.6 (40–50 °C)	
			6.7 (50–60 °C)	
			8.2 (60–70 °C)	
			11.0 (70–80 °C)	
			12.0 (80–90 °C)	
Balloon film	1.0 × 10^−3^	2.4 × 10^−4^	2.0	37

*Specific heat of the arterial wall, which is adjusted in our previous experiment,^24^ is reflected the thermal denaturation of collagen

We also found that the shape of SMCs was not changed after the conventional balloon dilatation. The sufficient arterial dilatation with the conventional balloon dilatation is obtained by the plastic deformation of the vessel wall. Many vascular injuries, such as tears, flaps, ruptures, and dissections, in the vessel wall were observed after the conventional balloon dilatation.^5^,^10^ The vessel wall divided into many sections due to the vascular injuries. This might be the reason why the SMCs’ shape is not changed, and we confirmed it in this study.

### Validity of the Method to Measure the Deformation Rate of SMCs’ Shape After Our Short-Duration Heating Dilatation by Cell’s Nuclei

**Figure 6:**
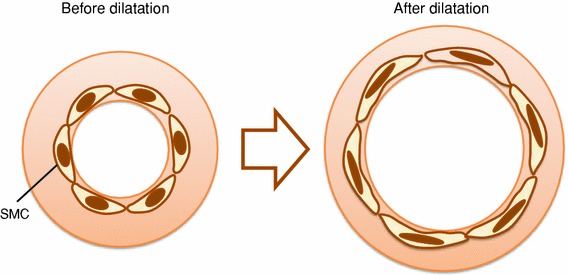
The schematic views: the deformation of the vessel wall and the SMCs after our short-duration heating dilatation

As long as the cross-sectional areas of the vessel wall before and after our short-duration heating dilatation are kept constant, the deformation rate of SMCs’ shape should have strong positive correlation with the transformation rate of the vessel wall, which is defined as dividing the ratio of the arterial diameter to the thickness of the vessel wall after dilatation by that before dilatation, because the SMCs, which are oriented low-angle spirally in the vessel wall,^1^ are stretched with the vascular wall due to the non-injured arterial dilatations with our short-duration heating dilatation (Fig. 6). The cross-sectional areas between internal elastic lamina and external elastic lamina before and after our short-duration heating dilatation, which were measured from the HE stained specimens, were almost same *ex vivo*: the ratios of areas after dilatations to before dilatations were less than 1.02. The ratios of the arterial diameter to the thickness of the vessel wall before and after our short-duration heating dilatation were measured, and the defined transformation rates of the vessel wall were calculated. We found that the correlation coefficient between the deformation rate of SMCs’ shape and the transformation rate was 0.99. Therefore, we conclude that there exists the correlation between the shape change of the SMCs and that of the SMCs’ nuclei, and thus, our measurement method for the deformation rate of SMCs’ shape is reasonable. In addition, we think that the measurement results of the deformation rates of SMCs’ shape are valid because of the validity of the measurement method.

### Comparison to Other Angioplasties

Hemostasis of the vessel wall is maintained by the SMCs in the vessel wall and the vasa vasorum around the arteries.^8^ According to reports of other thermal angioplasty and brachytherapy,^6^,^11^,^12^ significant decrease in the density of the SMCs in the vessel wall was observed after the dilatations. In addition, it is well known that the vasa vasorum was also injured by heating or γ-ray irradiation during other thermal angioplasty and brachytherapy. The restenosis rates on the chronic phase for other thermal angioplasty and brachytherapy were also reported to be high^17^,^19^,^22^ due to a lack of maintenance of hemostasis in the vessel wall after the dilatations.

Part of the SMCs was killed by short-duration heating^24^ and rest of the SMCs was alive with fixing in the stretched condition after our short-duration heating dilatation though the stretch stimulus might be severe damage to the SMCs. Although the deformation of the SMCs might be a negative factor in the chronic performance of our short-duration heating dilatation, no significant neo-intimal hyperplasia was observed after our short-duration heating dilatation *in vivo*. We prospect that the survival of part of SMCs is important to obtain sufficient arterial dilatation on the chronic phase despite the fixation of the SMCs in the stretched condition.

## Conclusions

We found that the SMCs in the vessel wall were fixed in the stretched condition after our short-duration heating dilatation and that this condition was maintained for 1 month in *in vivo* porcine study. We also demonstrated that the deformation rate of SMCs’ shape increased with rising *T*_balloon_. We hypothesize that the SMCs were fixed in the stretched condition because the arterial dilatation with our short-duration heating dilatation was performed without any plastic deformations of the vessel wall, causing the vessel wall itself to be stretched. We also prospect that the reasons for the positive correlation between the deformation rate of SMCs’ shape and *T*_balloon_ are that (i) the area heated over 60 °C was expanded with rising *T*_balloon_, and (ii) the arterial dilatation rate was also increased with rising *T*_balloon_.

## Abbreviation

SMC: Smooth muscle cell
